# Effects of the *Cistanche tubulosa* Aqueous Extract on the Gut Microbiota of Mice with Intestinal Disorders

**DOI:** 10.1155/2021/4936970

**Published:** 2021-07-14

**Authors:** Xiaowei Bao, Dongwen Bai, Xiaolu Liu, Ying Wang, Lanjun Zeng, Chenye Wei, Weiquan Jin

**Affiliations:** College of Food Science and Pharmacy, Xinjiang Agricultural University, Urumqi 830052, China

## Abstract

Disorders of the gut microbiota are associated with many diseases. The aqueous extract from *Cistanche tubulosa* (CT), a traditional Chinese herbal formula, has been reported to play a role in protecting the human intestine. However, little is known about its effects on the gut microbiota. The present study was carried out to determine whether the CT aqueous extract can modulate the gut microbiome in mice with intestinal disorders. We found that the damaged intestinal morphology resulting from treatment with cefixime could be rescued using the CT aqueous extract. The comparison of microbial diversity between mice treated with the CT extract and control mice also indicated that the disorder in the microbiome community of model groups could be restored by treatment with high and medium concentrations of the CT aqueous extract. Treatment with cefixime led to a significant decrease in lactic acid bacteria; however, the supplementation of the CT aqueous extract recovered the growth of these lactic acid bacteria. Furthermore, the CT aqueous extract was able to moderate the dramatic changes in the metabolic pathways of the gut microbiome induced by cefixime. These findings provided an insight into the beneficial effects of the CT aqueous extract on gut microbiota, and they also provided an important reference for the development of related drugs in the future.

## 1. Introduction

Intestinal microorganisms mainly colonize the intestinal lumen and mucosal layer and mutualize with the host through material and energy exchange, transformation, and other processes [1]. They are signaling hubs that integrate environmental messages, such as diet, with genetic and immune signals, consequently affecting the host's metabolism, immunity, nervous system, and response to infections [2]. Normally, there is a dynamic balance between the intestinal flora and hosts; however, gut dysbiosis can result in changes in health/disease balance, immune disorders, and a multitude of diseases [3]. Moderate changes in the gut microbiota are acceptable for the host; however, this can still provide opportunities to amplify the changes in other aggravating factors, such as bacteriophages, bacteriocins, and oxidative stress [4].

Previous studies have shown that the ethanol extract of *Cistanche tubulosa* (CT), a traditional Chinese herbal formula, can regulate the gut microbial composition in rats [5], and the total glycosides of CT adjusted the disordered gut microbiota [6]. *Cistanche* species, which mainly parasitize on the roots of *Tamarix* species, are also called “ginseng of the desert,” and a tonic consisting of the stems of *Cistanche deserticola* (CD) and *Cistanche tubulosa* (CT) is used as herbal remedy [7]. The main chemical components of CT phenylethanol glycosides (PHGs), which are antioxidant substances [8, 9], were found to improve reproductive dysfunction [10], suppress hepatic stellate cell activation, block the conduction of signaling pathways in TGF-*β*1/SMAD [11], and prevent bovine serum albumin-induced hepatic fibrosis in rats [12]. Among more than 100 components in CT, polysaccharide is also one of the important substances with abundant content [13, 14]. Previous studies have demonstrated that *C. deserticola* polysaccharides induce melanogenesis in melanocytes, reduce oxidative stress [15], alleviate cognitive dysfunction by regulating antioxidant and anti-inflammatory processes in rats [16], protect PC12 cells against OGD/RP-induced injury [17], enhance echinacoside absorption *in vivo*, and affect the gut microbiota [18].

Probiotics are live nonpathogenic microorganisms that have health benefits and confer microbial balance in the gastrointestinal tract when administered in adequate amounts [19]. They can enhance nonspecific cellular immune responses characterized by the activation of macrophages, natural killer (NK) cells, and antigen-specific cytotoxic T lymphocytes and the release of various cytokines in a strain-specific and dose-dependent manner [20]. Probiotic strains improve the properties of the intestinal epithelium via TJ modulation, and specific probiotic strains have been demonstrated to regulate mucin expression, thereby influencing the properties of the mucus layer and indirectly regulating the gut immune system [21]. Strains of lactic acid bacteria (LAB) and *Bifidobacterium* are major probiotics that have been used in many fields [22–26]. Their health benefits are numerous, with their antioxidant capacity being an important factor in their health-related functions [27]. Probiotics can chelate metal ions to prevent them from catalyzing oxidation [28, 29]; they can also increase the expression of antioxidant enzymes [30, 31], produce various metabolites with antioxidant activity [32, 33], mediate antioxidant signaling pathways [34–36], and regulate the enzymes producing reactive oxygen species (ROS) and the response of intestinal microorganisms to oxidative stress [37].

A recent study demonstrated that the polysaccharides of CD could stimulate the growth of some lactic acid bacteria, which could benefit human health [38]. However, the content of polysaccharides in CD is different from that in CT [7, 39], and this difference may lead to different effects on intestinal microorganisms. Furthermore, although CD polysaccharides can reduce oxidative stress by activating the NRF2/HO-1 pathway [15], the effects of single polysaccharide may differ from the overall effect of multiple compositions in CT. Thus, it is necessary to precisely define the effects of CT aqueous extracts on intestinal microorganisms. In addition, PHGs can also resist oxidative stress [40] and suppress lipopolysaccharide-mediated inflammatory responses by activating the Keap1/Nrf2/HO-1 pathway [41]. Therefore, determining the effect of the CT aqueous extract is of great value. In addition, the effects of certain constituents of the aqueous CD extract on oxidative stress and intestinal flora suggest that the resistance to oxidative stress might be correlated with intestinal flora changes.

In order to fill the gaps in the knowledge on the topics mentioned above, we investigated the effects of the CT aqueous extract on the gut microbiota of mice with intestinal flora disorders. These results will provide valuable information about the possible mechanisms through which CT changes the intestinal flora and confers gut resistance to oxidative stress.

## 2. Materials and Methods

### 2.1. Experimental Animals

A total of 18 SPF-class male C57BL/6J mice, weighing 18–22 g, were purchased from the Experimental Animal Center of Xinjiang Medical University with license number SCXK (new) 2018-0003. They were housed in cages under standardized conditions: 12 h light/dark photoperiod, temperature of 23 ± 2°C, and humidity of 55 ± 5%. The animals were fed a commercial diet (51% nitrogen-free extract, 25% crude protein, 4.6% crude fat, 6.5% crude ash, 4.0% crude fiber, and 8.9% moisture) and tap water. The animals were treated according to the recommendations described in the Guide for the Care and Use of Laboratory Animals of the National Institutes of Health.

### 2.2. Extraction of the Aqueous Extract

Dried slices of *C. tubulosa*, provided by Hotan Dichen Pharmaceutical Biotechnology Co., Ltd., were ground into powder, and granules with particle sizes between 20 and 40 meshes were selected. The extraction conditions were as follows: solid-liquid ratio of 1 : 19, temperature of 80°C, microwave time of 6 min, ultrasonic time of 16 min, microwave power of 400 W, and ultrasonic power of 400 W. The contents of the main components of the aqueous extract were measured by HPLC (Agilent 1260 Infinity II, California, USA). In brief, the standard substances of echinacoside (0.2 mg/mL) and acteoside (0.2 mg/mL) were dissolved in 50% methanol to serve as reference substance solution. Then, 1 g of the CT aqueous extract was dissolved in 100 mL 50% methanol and left to set for 30 min. The extract solution was treated with ultrasound at 250 W and 35 kHz for 10 min and subsequently centrifuged at 12,000 rpm/min. The supernatant was filtrated by a 0.45 *μ*m microporous filter membrane. The reference substance solution and the filtrate were then detected by HPLC in the following conditions: octadecylsilane-bonded silica gel as filler, methanol as mobile phase A, and 0.1% formic acid as mobile phase B. The temperature of the column was set as 30°C, the detection wavelength was set as 330 nm, and the injection volume was 10 *μ*L.

### 2.3. Experiments

After one week of adaptation, the 18 mice were randomly divided into six groups: A (normal with middle-dose CT aqueous extract added), B (normal without the CT aqueous extract), C (model without the aqueous extract), D (model with high-dose CT aqueous extract added), E (model with middle-dose CT aqueous extract added), and F (model with low-dose CT aqueous extract added). The groups were treated as follows: the normal group was drenched by normal saline solution, model group was drenched by cefixime (30 mg/kg, Shiyao Group Ouyi Pharmaceutical Co., Ltd., Shijiazhuang, China) and normal saline solution, high-dose group was drenched by cefixime and 221.14 mg/kg of the CT aqueous extract, middle-dose group was drenched by cefixime and 165.54 mg/kg of the aqueous extract, and low-dose group was drenched by cefixime and 110.57 mg/kg of the aqueous extract. The A group was drenched with 165.54 mg/kg of the aqueous extract, and no cefixime was added. Cefixime was administered daily at 12:00 h, and other substances were administered daily at 15:00 h. During the experiments, the C, D, E, and F groups were kept in the model state of intestinal disorders. The feces were collected every seven days on a sterile operable table and stored at −20°C.

### 2.4. Histopathological Observation of the Mice Colon

At the end of the experiment, the mice were killed by cervical dislocation, and their colon contents were collected on a sterile operable table and stored at −80°C; at the same time, colonic tissue samples were fixed in 10% neutral formalin. Then, the samples were dehydrated using gradient concentration of ethanol, hyalinized using xylene, embedded in paraffin, sectioned, and stained with hematoxylin-eosin. Morphological changes in the colonic mucosa were observed and compared using an optical microscope. Villus length and crypt depth in the colon were measured, and the ratio of villus length to crypt depth (V/C value) was calculated (51).

### 2.5. DNA Extraction and Library Construction

DNA was extracted from the feces using the E.Z.N.A. ®Soil DNA Kit (Omega Bio-Tek, Norcross, GA, USA) according to the manufacturer's protocol. DNA quality was determined using a fluorometer (QuantiFluor™–ST, Promega Corporation, USA). Paired primers in the V3-V4 region of 16s rDNA were designed to amplify the region and produce 466 bp DNA fragments. The forward primer was 341F (-5-CCTACGGGNGGCWGCAG-3-), and the reverse primer was 806R (-5-GGACTACHVGGGTATCTAAT-3-). Each PCR volume was 25 *μ*L, containing 2.5 *μ*L of 10 × PCR buffer, 2 *μ*L of dNTPs, 1 *μ*L of each primer, and 20–30 ng of template DNA. Then, the indexed adapters were attached to the end of the amplicons to generate sequencing libraries. The libraries were validated using a QuantiFluor™ fluorometer and quantified to 10 nmol.

### 2.6. 16s rRNA Gene Sequencing and Microbial Community Analysis

The Illumina platform (Illumina MiSeq) was used to obtain 2 × 250 bp paired-end data. Operational taxonomic units (OTUs) were obtained using Uparse software through standard clustering with 97% similarity. The naive Bayesian assignment algorithm of the RDP classifier was used to align the OTUs with the Greengene database Release 13.5 and perform species annotation. The alpha diversity of gut microbiota was calculated using the Shannon and Simpson indices, and the differences between groups were analyzed by Linear discriminant analysis Effect Size (LEfSe). The beta diversity was analyzed by principal coordinate analysis (PCoA) of Bray–Curtis dissimilarities. PICRUSt2 was used to estimate the microbial metabolic capacity of the gut microbiome [42].

### 2.7. Statistical Data Analysis

SPSS 20 was used for one-way ANOVA, and the experimental data were expressed as *X* ± *S*; *X* indicates the average value, and *S* indicates the standard deviation.

## 3. Results

### 3.1. The Effect of the CT Aqueous Extract on Colon Morphology

**Figure 1:**
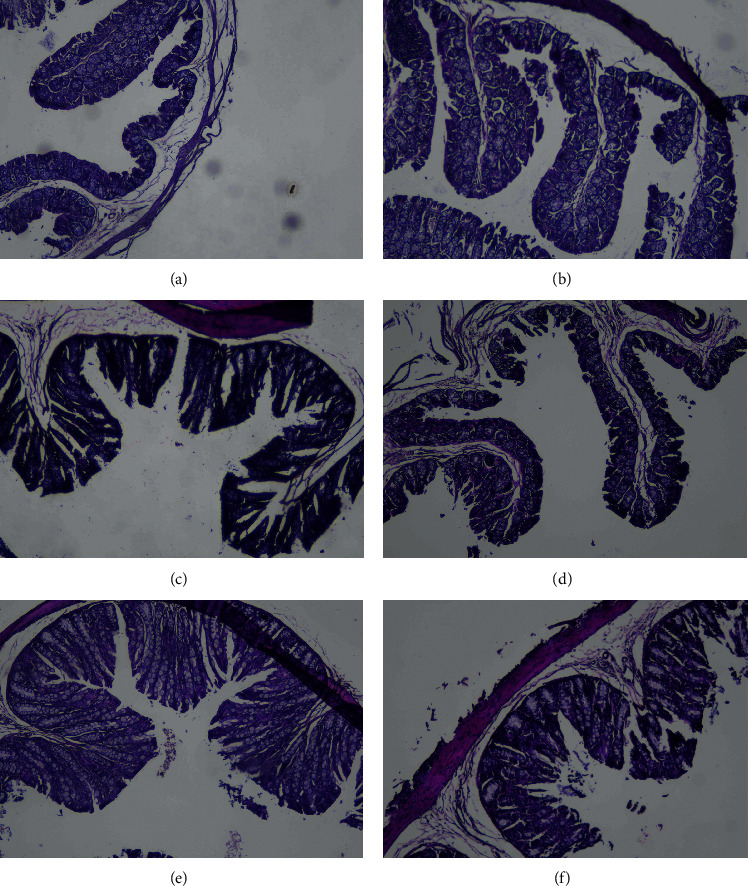
The effect of the aqueous *Cistanche tubulosa* (CT) extract on colon morphology: A, normal group with middle-dose aqueous *Cistanche tubulosa* (CT) extract added; B, normal group; C, model group; D, model group with high-dose CT aqueous extract added; E, model group with middle-dose CT aqueous extract added; F, model group with low-dose CT aqueous extract added. (a) Group A. (b) Group B. (c) Group C. (d) Group D. (e) Group E. (f) Group F.

The representative compounds (echinacoside and acteoside) and their concentrations of the CT extract were validated by HPLC (Figure S1). To determine the effect of the aqueous extract on the gut, we investigated the length of colon villi and depth of recessus following the treatment with the CT aqueous extract. The colon villi in the normal and high-dose groups (A, B, and D) were longer and fingerlike, whereas the colon villi in the model and low-dose groups (C and F) were short, and the tips of the colon villi were broken (Figure 1). Accordingly, high-dose CT aqueous extract significantly increased the length of colon villi and reduced recessus depth in mice with intestinal disorders compared with the mice in the model group (*P* < 0.01). In contrast, recessus depth was not significantly different between the high-dose group and normal group (*P* > 0.05) (Table S1). These results indicated that the high dose of the CT aqueous extract can improve the morphology inside the colon of mice with intestinal disorders.

### 3.2. The Effect of the CT Aqueous Extract on the Diversity of Gut Microbiota

**Figure 2:**
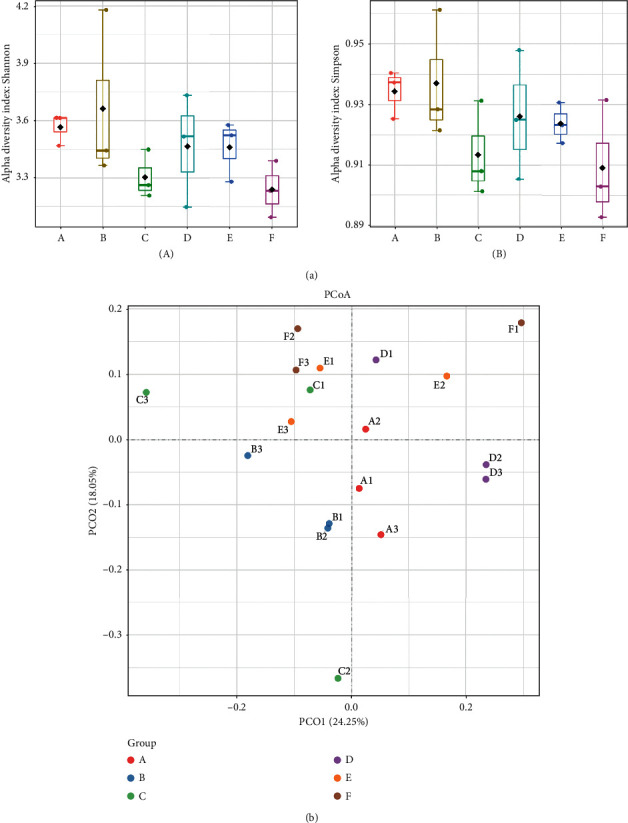
Gut microbiota diversity analysis of mice feces. (a) *α*-Diversity of bacteria communities measured by the Shannon index (A) and the Simpson index (B); (b) the principal coordinate analysis (PCoA) plot visualizing the data based on Bray–Curtis dissimilarities. A, normal group with middle-dose aqueous *Cistanche tubulosa* (CT) extract added; B, normal group; C, model group; D, model group with high-dose CT aqueous extract added; E, model group with middle-dose CT aqueous extract added; F, model group with low-dose CT aqueous extract added.

We performed 16s rRNA gene sequencing to investigate the potential cause of the morphological changes inside the colon and investigate the changes in gut microbiota following treatment with the CT aqueous extract. An average of 100,553 effective tags, ranging from 77,734 to 125,144, was obtained from the raw data (Table S2). These tags were clustered into 4932 OTUs (Table S3). We then analyzed the diversity of the gut microbiota based on these OTUs. The Shannon and Simpson indexes showed no difference between the A group (normal with the CT aqueous extract) and B group (normal without the CT aqueous extract) (Figure 2(a)). This indicated that, in the mice without the cefixime treatment, the CT aqueous extract might have had no additional beneficial or harmful effects on the *α*-diversity of the gut microbiota. However, the *α*-diversity in the model group (C) showed a decreasing trend compared to that in the normal groups. The mice treated with high- and middle-dose CT aqueous extracts showed signs of *α*-diversity recovery, whereas such a phenomenon was not observed in mice treated with the low-dose CT aqueous extract (Figure 2(a)). Meanwhile, the PCoA revealed that the normal groups (A and B) and intestinal disorder groups administered high-dose (D) and middle-dose (E) CT aqueous extracts tended to have shorter intersample distances than those in the model group and in the low-dose CT aqueous extract supplement group (F) (Figure 2(b)). These results indicated that the CT aqueous extract could help improve the diversity of the gut microbiota in mice with intestinal disorders.

### 3.3. Changes in the Composition of Gut Microbiota Treated with the CT Aqueous Extract

**Figure 3:**
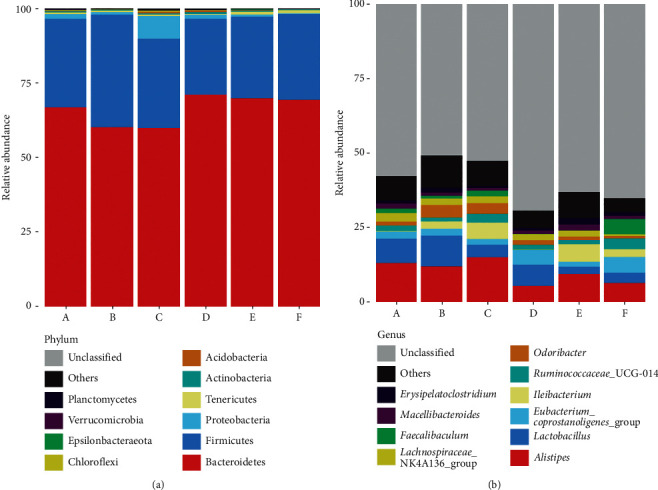
The composition and relative abundance of gut microbiota in mice feces at the phylum and genera levels. (a) Phylum level classification composition and relative abundance. (b) Genera level classification composition and relative abundance. A, normal group with middle-dose aqueous *Cistanche tubulosa* (CT) extract added; B, normal group; C, model group; D, model group with high-dose CT aqueous extract added; E, model group with middle-dose CT aqueous extract added; F, model group with low-dose CT aqueous extract added.

The microbiota composition profiles were compared among different groups. At the phylum level, the relative abundance of Proteobacteria in the model group was higher than that in the other groups (Figure 3(a)). The increase in Proteobacteria suggested that the microbiome of model mice was changed by cefixime and that the CT aqueous extract might benefit the intestinal microbiota as the increased prevalence of Proteobacteria is a hub marker of disordered intestinal flora [43–45]. In addition, at the genus level, the relative abundance of *Lactobacillus* in the model group decreased compared with that in the normal and high-dose groups; however, it increased compared with that in the middle- and low-dose group (Figure 3(b)). These results indicated that the high-dose CT aqueous extract might promote the growth of some bacteria from the genus *Lactobacillus*.

**Figure 4:**
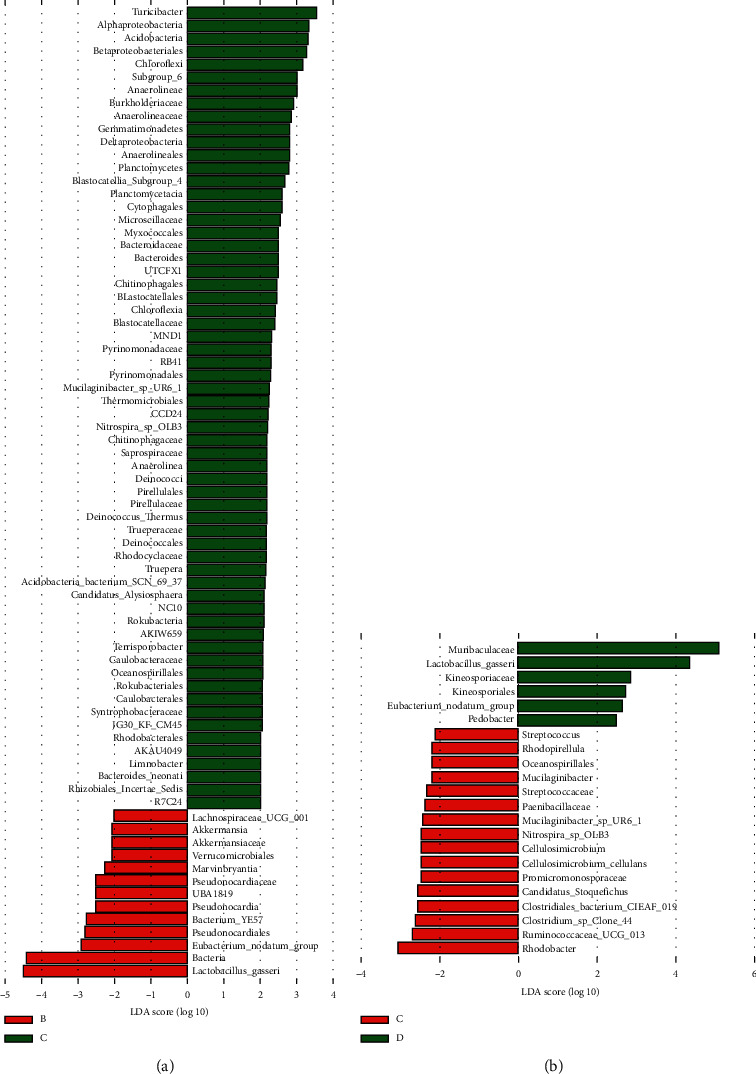
Results of the LEfSe analysis between different groups. (a) Relative abundance of bacteria between the normal group (B) and model group (C); (b) relative abundance of bacteria between the model group (C) and model group with high-dose aqueous *Cistanche tubulosa* (CT) extract added (D).

Differential microbiota between the studied groups were further determined according to the LEfSe analysis. This analysis showed that, after the treatment with cefixime, the relative abundances of *Turicibacter*, Alphaproteobacteria, Acidobacteria, Betaproteobacteriales, and Chloroflexi significantly increased, whereas the relative abundances of *Lactobacillus*, *Eubacterium_nodatum_*group, Pseudonocardiales, and Christensenellaceae_R-7_group significantly decreased compared with those in the normal group (Figure 4(a)). Strikingly, when the model group was supplemented with the high-dose CT aqueous extract, the relative abundances of *Muribaculaceae*, *Lactobacillus*, Kineosporiaceae, *Eubacterium nodatum* group, and *Pedobacter* were significantly increased compared to those in the model group. Meanwhile, the relative abundances of *Rhodobacter*, Ruminococcaceae UCG_013, *Roseburia*, *Ruminiclostridium_9*, and *Candidatus Stoquefichus* decreased significantly compared to those in the model group (Figure 4(b)).

### 3.4. Functions of the Gut Microbiota Related to the Treatment with the CT Aqueous Extract

**Figure 5:**
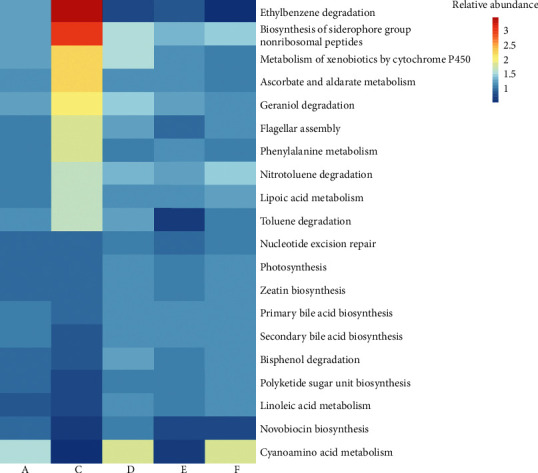
Changes in the relative abundance of metabolic pathways in the normal group with middle-dose aqueous *Cistanche tubulosa* (CT) extract added (A), model group (C), model group with high-dose CT aqueous extract added (D), model group with middle-dose CT aqueous extract added (E), and model group with low-dose CT aqueous extract added (F). The abundances are normalized according to the values in the normal group (B). Color depth indicates the magnitude of the ratio.

We used PICRUSt2 software to predict the metabolic pathways of the gut microbiota, and the normal group was used as a reference to analyze the changes in other groups. Under the cefixime treatment, the relative abundance of ethylbenzene degradation, biosynthesis of siderophore group nonribosomal peptides, and metabolism of xenobiotics by cytochrome P450 pathways increased; after the treatment with high- and middle-dose CT aqueous extracts, their relative abundance returned to normal levels. Meanwhile, the relative abundance of the cyanoamino acid metabolism pathway decreased under the cefixime treatment; however, it increased after the treatment with the high-dose CT aqueous extract. Furthermore, in general, the changes in different metabolite pathways after the treatment with cefixime were significant compared with those in the normal group; however, the addition of the CT aqueous extract was able to prevent excessive changes (Figure 5).

## 4. Discussion

Colon morphology can be altered by growth, digestion and absorption, immune regulation, and intestinal injury repair [46–50]. The V/C ratio can comprehensively reflect the digestive status of the intestinal tract and is directly proportional to the digestive and absorption capacity of the gut tract [51, 52]. In the present study, the villi and recessus biopsy and statistical data showed that the high-dose aqueous extract could partly improve the defective morphology inside the colon.

To investigate how the aqueous extract changes the gut morphology and affects the intestinal microbiota, we worked backward from changes in the intestinal flora. We found that the relative abundance of Proteobacteria, a hub marker of disordered intestinal flora, increased under the treatment with cefixime compared to that without cefixime treatment. The relative abundances of other hub markers, Bacteroidetes and Firmicutes, had no significant changes, although these groups are predominant in the human gut; the ratio of Bacteroidetes/Firmicutes was found to be decreased in obese people compared with that in lean people, and this ratio was found to increase with weight loss in people on two types of low-calorie diet [38, 41, 43–45, 48, 53, 54]. Meanwhile, *Turicibacter*, which is associated with obesity [55], was significantly elevated in the model group compared to that in the other groups. Notably, the diversity of gut microbiota in the model mice was improved by the addition of the CT aqueous extract. We noted some specific intestinal bacteria in mice under different treatments; for example, *Lactobacillus* and *Muribaculaceae* were the two main bacterial genera that increased in the group treated with the high-dose CT aqueous extract compared to those in the model group (Figure 4). Recent studies have indicated that the polysaccharides of CT aqueous extracts possess significant antioxidant activities *in vitro* [56] and can promote the growth of some lactic acid bacteria, which could benefit host health [43]. In parallel, *Muribaculaceae* are probiotic organisms linked to longevity [57]. These suggested that the mechanism by which the CT aqueous extract improves the gut microbiota may be the promotion or protection of the growth of probiotic organisms. Another bacterium worthy of note was bacterium YE57. Although the high-dose CT aqueous extract promoted the relative abundance of bacterium YE57 in the present study (Figure 4), previous studies have found that its abundance was higher in the normal gut than that in the gut treated with high-concentration herbal tea residue [58] and that its abundance was reduced after the intervention with *Bacillus licheniformis* combined with XOS (xylooligosaccharides) [59]. Thus, the role of this bacterium in the gut microbiota deserves further study. Besides, the relatively small sample number in this study might cause a measure of false positive and false negative, and future study on larger samples is suggested to validate the identified bacterial markers.

CT aqueous extract composition might be important for its effects on the composition and functional changes in the gut microbiota of mice with intestinal disorders. PHGs are common active components found in CD and CT, and echinacoside was identified as the major PHG in CT [60]. In the past decades, echinacoside has been shown to possess many pharmacological activities, such as antiaging and neuroprotective effects, improvement of cardiac function, reduction of hyperlipidemia and hyperglycemia, and prevention of obesity-induced diabetes and metabolic syndrome [53, 61–65]. In fact, we detected changes in the metabolic pathways of the gut microbiota. The treatment of cefixime led to the enrichment of bacteria related to ethylbenzene degradation and biosynthesis of siderophore group nonribosomal peptides, while the treatments with the high- and middle-dose CT aqueous extract could alleviate these changes, indicating that this extract moderated the bacterial community related to these functions. In addition, the increased bacterial enrichment related to the cyanoamino acid metabolism pathway under the treatment with the high-dose aqueous extract and its decreased enrichment in model mice indicated that the CT aqueous extract can promote the metabolism of cyanoamino acid. The changes in relevant metabolites might provide this aqueous extract with pharmacological activities.

Although the mechanism by which the CT aqueous extract changes the composition and function of intestinal microbiota is complex, there are some clues to speculate about the potential mechanism. It has been reported that both lactic acid bacteria and CT aqueous extracts can antagonize oxidative stress. Oxidative stress occurring during inflammation is a common factor that exacerbates intestinal disorders by strongly reducing gut microbial diversity and promoting the surge of specific bacteria (4). On the contrary, reactive oxygen species also promote the selective growth of bacterial groups through nitrate and tetrathionate respiration [66–68]; for example, bacteria from the family Enterobacteriaceae can grow rapidly as a consequence of changes in the composition of the intestinal flora under oxidative conditions during inflammation [69, 70]. Most living organisms evolve enzymatic defenses, nonenzymatic antioxidant defenses, and repair mechanisms to scavenge oxygen radicals [71]. However, these native antioxidant systems are generally not sufficient to prevent oxidative damage in living organisms. Several additional synthetic antioxidants, including butylated hydroxyanisole and butylated hydroxytoluene, have been widely used to decrease oxidation, but their safety has been questioned [72, 73]. Therefore, researchers have turned to finding safer and more natural antioxidants obtained from naturally occurring substances. Due to the ability of both polysaccharides and lactic acid bacteria to eliminate oxidative stress, determining the precise antioxidative mechanism of CT aqueous extracts on the intestinal microbiota requires further investigation in the future.

In conclusion, we found that the CT aqueous extract was able to improve intestinal gut microbiota in mice with intestinal disorders by promoting the diversity, moderating the metabolic changes, and remodeling the structure of gut microbiota, and these results may provide a reference for the development of related drugs in the future.

## Data Availability

The statistic of the operational taxonomic units among each sample data used to support the findings of this study are included within the supplementary information files, and the 16s rRNA sequencing data used to support the findings of this study will be released upon publication.

## Supplementary Materials

Supplementary Materials Figure S1: HPLC detection of the *Cistanche tubulosa* aqueous extract. (a) The peak of echinacoside and acteoside in the reference material appears at 5.066 min and 9.988 min separately. (b) The peak of echinacoside and acteoside in the aqueous extract appears at 5.097 min and 10.076 min separately, and the concentrations are 236 mg/g and 12.7 mg/g separately. Table S1: the length of colon villi and depth of recessus. Table S2: statistic information of the 16S rRNA sequencing data. Table S3: statistic of the operational taxonomic units among each sample.
