# Supplementary material for: Effects of the Cistanche tubulosa Aqueous Extract on the Gut Microbiota of Mice with Intestinal Disorders
Source: Evid Based Complement Alternat Med. 2021 Jul 14;2021:4936970. doi: 10.1155/2021/4936970 (PMC8294959; doi:10.1155/2021/4936970)
Supplement: Supplementary Materials — Figure S1: HPLC detection of the Cistanche tubulosa aqueous extract. (a) The peak of echinacoside and acteoside in the reference material appears at 5.066 min and 9.988 min separately. (b) The peak of echinacoside and acteoside in the aqueous extract appears at 5.097 min and 10.076 min separately, and the concentrations are 236 mg/g and 12.7 mg/g separately. Table S1: the length of colon villi and depth of recessus. Table S2: statistic information of the 16S rRNA sequencing data. Table S3: statistic of the operational taxonomic units among each sample. [file 4936970.f1.zip › 4936970.f1/SUPPLEMENTARY DESCRIPTION.docx]

**SUPPLEMENTARY DESCRIPTION:**

**Figure legends**

**Figure S1.** HPLC detection of *Cistanche tubulosa* aqueous extract. (a) The peak of echinacoside and acteoside in reference material appear at 5.066 min and 9.988 min separately. (b) The peak of echinacoside and acteoside in aqueous extract appear at 5.097 min and 10.076 min separately, and the concentrations were 236 mg/g and 12.7 mg/g separately.

**Table S1**. The length of colon villi and depth of recessus.

**Table S2.** Statistic information of 16S rRNA sequencing data.

**Table S3.** Statistic of the operational taxonomic units among each sample.
