# Supplementary material for: Effects of the Cistanche tubulosa Aqueous Extract on the Gut Microbiota of Mice with Intestinal Disorders
Source: Evid Based Complement Alternat Med. 2021 Jul 14;2021:4936970. doi: 10.1155/2021/4936970 (PMC8294959; doi:10.1155/2021/4936970)
Supplement: Supplementary Materials — Figure S1: HPLC detection of the Cistanche tubulosa aqueous extract. (a) The peak of echinacoside and acteoside in the reference material appears at 5.066 min and 9.988 min separately. (b) The peak of echinacoside and acteoside in the aqueous extract appears at 5.097 min and 10.076 min separately, and the concentrations are 236 mg/g and 12.7 mg/g separately. Table S1: the length of colon villi and depth of recessus. Table S2: statistic information of the 16S rRNA sequencing data. Table S3: statistic of the operational taxonomic units among each sample. [file 4936970.f1.zip › 4936970.f1/Table_S2.pdf]

Table S2. Statistic information of 16S rRNA sequencing data

SampleID	Total Tags	Unique Tags	Taxon Tags	Singleton Tags	OTUs
A1	96824	57827	81643	15181	1306
A2	85295	51216	73859	11436	1496
A3	89389	54314	75235	14154	1278
B1	89452	52879	73610	15842	943
B2	87138	54837	71106	16032	1241
B3	88442	51958	75367	13075	1196
C1	92360	54950	77953	14407	1318
C2	77734	47354	66510	11224	1131
C3	88245	50988	81792	6453	2138
D1	114285	64211	94944	19341	1535
D2	111186	63146	95744	15442	2068
D3	106642	61205	88255	18387	1320
E1	125144	71712	105996	19148	1746
E2	96093	56787	85438	10655	1300
E3	105541	60131	88235	17306	891
F1	119613	68024	101475	18138	1035
F2	123561	63729	107787	15774	1032
F3	113014	64280	98882	14132	1143
Avg	100553	58308	85768	14784	1339
